# Digital Orthodontics: Posterior crossbite correction and arch expansion with directly 3-dimensional printed clear aligners

**DOI:** 10.4317/jced.63389

**Published:** 2025-12-30

**Authors:** Jae Hyun Park, Miyoung Sim, Hyewon Choi, Hyun-Hee Choo

**Affiliations:** 1 Postgraduate Orthodontic Program. A.T. Still University-Arizona School of Dentistry &amp; Oral Health. 5835 E. Still Circle Mesa, AZ 85206; 2 Orthodontic &amp; Craniofacial Development Research. ADA Forsyth Institute. 100 Chestnut. Sommerville, MA 02143; 3 Division of Orthodontics. College of Dentistry. The Ohio State University. 305 West 12th Ave. Columbus, OH 43210; 4 Department of Orthodontics. Gwangmyeong Hospital Chung-Ang University. Gwangmyeongsi Kyeonggido, South Korea

## Abstract

Posterior crossbite correction and mandibular arch expansion have traditionally been managed with fixed appliances, while conventional thermoformed aligners have shown limited efficacy due to material properties and biomechanical constraints. This case report describes the management of bilateral premolar crossbite and mandibular constriction in a 26-year-old female using directly 3D-printed clear aligners (DPA) fabricated with a biocompatible resin. A complete digital workflow treatment planning was employed, and twenty-six aligners were produced using high-resolution LCD printing over a treatment period of thirty-one weeks. Posttreatment evaluation confirmed successful correction of the crossbite, stable arch expansion, and maintenance of posterior inclination control. Three-dimensional superimposition and colormap analysis demonstrated minimal relapse, less than 0.5 mm, at one-year follow-up. This report highlights the potential of DPA as a clinically effective alternative to thermoformed aligners for the correction of complex occlusal discrepancies.

## Introduction

Since Invisalign introduced clear aligner therapy in the 1990s, aligners, traditionally fabricated from thermoplastic sheets vacuum-pressed over 3D-printed sequential dental models, have become a popular and effective alternative to fixed appliances for esthetic purposes (1). Recent advances in CAD/CAM technology and photopolymer materials have led to the development of directly 3D-printed clear aligners (DPA), which are designed directly over digital models and then 3D-printed using light-cured, biocompatible resins. Direct printing eliminates the need for an intermediate model, allowing software-controlled adjustment of aligner thickness and shape according to each shell region. Hence, this method simplifies the workflow, saves time and cost, enhances mechanical efficiency by offering enhanced precision and accuracy, and reduces environmental waste (2). Recently, an increasing number of studies have reported enhanced manufacturing productivity, ensuring biosafety as well as validating the biomechanical efficacy of DPA systems (3 - 5). Previous clinical reports on DPA have largely focused on anterior teeth alignments (6 - 8). This case report demonstrates the novel and successful application of DPA to more complex movements, namely bilateral premolar crossbite correction and mandibular arch expansion. It also evaluates the stability of these outcomes one-year posttreatment.

## Case Report

**Figure 1:**
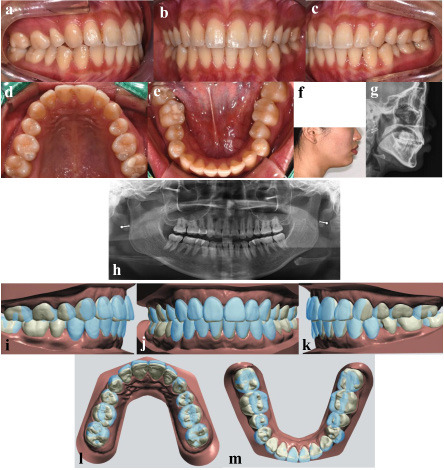
Diagnosis and virtual treatment plan. Pretreatment intraoral and facial photos, and cephalometric and panoramic radiographs (a-h). Treatment setup (i-m); Pretreatment occlusion is shown in blue, and the planned occlusion is shown in white.

A 26-year-old female presented with the chief complaint that the misalignment of her mandibular arch was visible when smiling. She expressed a strong preference for aligner therapy over fixed appliances. Her orthodontic history included extraction of her upper first premolars during comprehensive treatment with fixed appliances when she was an adolescent, followed by inadequate retainer wear, which led to relapse. Clinical examination revealed bilateral crossbites of the mandibular first premolars, an overjet of 3.1 mm, an overbite of 1.8 mm, quarter-cusp Class II canine relationships bilaterally, and full cusp Class II molars (Fig. 1a-e).

1

Cephalometric analysis indicated a mild skeletal Class II pattern with an ANB angle of 4.2° and a high mandibular plane angle of 30.9° (Fig. 1g, Table 1).Table 1The treatment objectives were to correct the bilateral premolar crossbites and achieve arch coordination through dentoalveolar expansion, all within the treatment period of less than one year, in accordance with the patient's preference for a quick retreatment.

**Figure 2:**
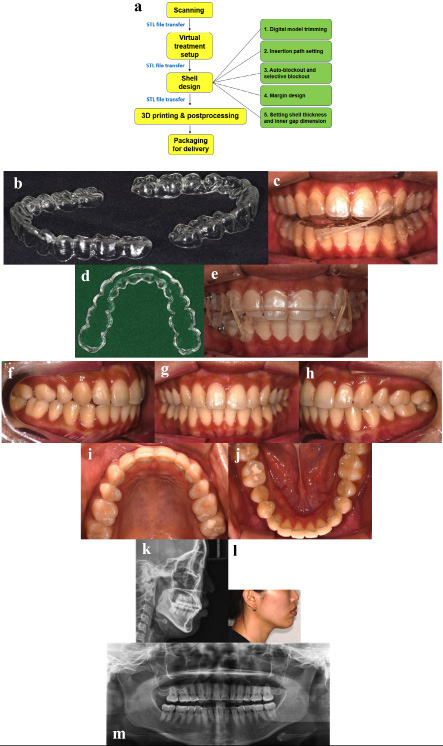
Appliance Design, and treatment progress and results. Flow of design and production of Direct Print Aligner (a-b). Patient is wearing the midline elastic (c), and box elastics in the lower row with wrap-around type aligner (d,e). Treatment results (f-m).

Digital impressions were obtained using a TRIOS 3 (3Shape, Copenhagen, Denmark), and virtual treatment planning was performed with the 3Shape Ortho System, an orthodontic CAD software (Fig. 1i-m). The setup prescribed up to 0.2 mm of translation and 2° of rotation per stage. Aligner shells were designed with a thickness of 0.5 mm and an inner gap of 30 m, incorporating selective blockouts to streamline the force delivery (Fig. 2a).

2

The aligners were printed with FDA-cleared and biocompatible clear resin (ODS Co., Ltd., Incheon, South Korea) using an 8K LCD printer (Sonic Mini 8K; Phrozen Tech Co., Hsinchu, Taiwan) and then underwent standardized post-processing UV-light and heat curing (Fig. 2b) (7).

The patient wore each aligner for approximately twenty hours per day, progressing to the next in sequence every seven days. After seventeen weeks, the crossbite had resolved, and mild distal movement of the maxillary canines was noted. Cross-arch elastics were added to assist with midline refinement (Fig. 2c). Upon completion of the initial twenty-five aligners, slight open bite in the right posterior region and small residual spaces prompted the fabrication of refinement aligners. The aligners were designed as wrap-around types with vertical and posterior box elastics, allowing for occlusal settling without unwanted molar intrusion (Fig. 2d,e).

**Figure 3:**
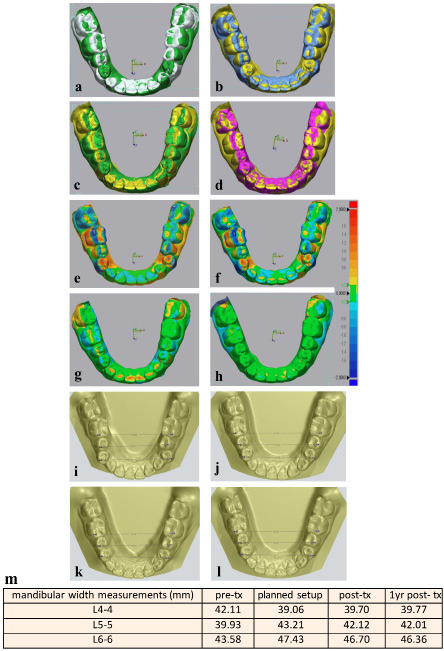
Treatment outcome. Best fit superimposition of lower arch by Geomagic control X (a-d); a, Pretreatment in grey and planned setup in green. b, Pretreatment in blue and posttreatment in yellow. c, Planned setup in green and posttreatment in yellow. d, Posttreatment in yellow and one-year follow-up in violet. Superimposed 3D colormaps by Geomagic control X (e-h); e, Pretreatment and planned setup. f, Pretreatment and posttreatment. g, Planned setup and posttreatment. h, Posttreatment and one-year follow-up. Mandibular width measurements (i-m); i, Pretreatment. j, Planned setup. k, Posttreatment. l, One year follow-up. m, Summary of mandibular width measurements.

The active treatment phase was completed in thirty-one weeks using twenty-six aligners. The bilateral premolar crossbites were corrected, and the arches were well coordinated. Fixed lingual retainers were bonded, and night-time DPA retainers were delivered (Fig. 2f-m). The treatment progressed without discomfort or complications, consistent with the limited treatment objectives. Best-fit superimposition with Geomagic Control X demonstrated increased mandibular arch widths and corrected posterior buccolingual crown inclinations from pre- to posttreatment (Fig 3a-d).

3

Three-dimensional color maps corroborated high treatment efficacy with minimal relapse of the lower arch (Fig. 3e-h). Mandibular inter-tooth width measurements confirmed expansion at the second premolar and first molar levels and effective correction of the first-premolar buccal crossbite, all maintained within 0.5 mm of change at one year (Fig. 3i-m).

## Discussion

This report highlights the clinical ability of DPA to correct premolar crossbites and achieve dentoalveolar arch expansion, which, to our knowledge, have not been previously reported in clinical cases. The performance of thermoformed aligners has been inferior to fixed appliances in correcting posterior crossbites and achieving expansion (1 , 9 - 12). Evidence indicates a predominance of tipping movements with reduced posterior efficacy, necessitating overcorrection and multiple attachments (13 - 15). DPA represents a significant advance in aligner therapy, attributed to its unique resin properties and its ability to deliver precise biomechanical forces with less attachments. An in vitro comparison of DPA resins and thermoplastics showed that DPA with localized thickness modifications exhibited a broader range of compressive strength, supporting targeted force application (14). Unlike thermoformed aligners which generate high initial forces that rapidly diminish, DPA maintains more consistent force levels (5). Their combination of firmness and elasticity allows secure engagement of undercut areas and effective retention without bonded attachments (7). DPA resin's shape-memory feature provides a unique biomechanical advantage. When immersed in water above 60 °C, the aligners regain their original shape, restoring accurate fit even after prolonged use (PTC patent application No. PTC/KR2024/021101) (7 , 15). In addition to biomechanical benefits, ODS clear resin has been approved by the U.S. Food and Drug Administration (FDA) and the European Commission (EC), and validated through in vivo and in vitro cytotoxicity testing (7). Its low viscosity and high mechanical strength enable fabrication of aligners as thin as 0.5 mm, while also allowing the integration of structural modifications such as elastic buttons (Fig. 2b,c). Continued investigation of DPA will expand aligner therapy into a broader range of clinical applications within digital orthodontics. In conclusion, DPA effectively corrected bilateral premolar crossbites and mandibular constriction through dentoalveolar expansion, with results stable at one-year follow-up. The advantages in design flexibility, force control, and manufacturing efficiency suggest that DPA may be a valuable tool in managing malocclusions which have traditionally been less responsive to thermoformed aligners.

## Figures and Tables

**Table 1:** Cephalometric summary.

Measurement	Norm	Pretreatment	Posttreatment
SNA (°)	81.1±3.7	79.9	79.9
SNB (°)	79.2±3.8	75.7	75.7
ANB (°)	2.5±1.8	4.2	4.2
U1-SN (°)	105.3±6.6	101.9	102.9
FMIA (°)	65.0±5.0	56.8	56.8
FMA (°)	25.0±4.0	30.9	30.9
IMPA (°)	90.0±3.5	92.3	92.4
Overbite (mm)	2.0±2.0	1.8	1.7
Overjet (mm)	2.0±2.0	3.1	3.0

SNA, sella-nasion-A point; SNB, sella-nasion-B point; ANB, A point-nasion-B point; U1-SN, maxillary incisor to sella-nasion; FMIA, mandibular incisor to Frankfort horizontal plane; FMA, gonion-menton to Frankfort horizontal plane; IMPA, mandibular incisor to gonion-menton.

## Data Availability

The datasets used and/or analyzed during the current study are available from the corresponding author.
